# Geographical distribution and social determinants of Tobacco 21 policy adoption and retail inspections in the United States, 2015–2019

**DOI:** 10.18332/tid/140148

**Published:** 2021-09-16

**Authors:** Hongying Dai, Niran Tamrakar, Nirosha Rathnayake, Kaeli Samson

**Affiliations:** 1 College of Public Health, University of Nebraska Medical Center, Omaha, United States

**Keywords:** social determinant, disparities, Tobacco 21

## Abstract

**INTRODUCTION:**

Tobacco control laws that raise the minimum age of tobacco sales to 21 years (T21) play a pivotal role in youth tobacco prevention, yet empirical data are sorely needed to inform enforcement, compliance efforts, and future legislation.

**METHODS:**

Spatial analysis was conducted at the zip code level by geocoding the states and localities that adopted T21 ordinances from 2015 to 2019. A multi-level logistic regression model was conducted to examine disparities in neighborhood socioeconomic status (SES), FDA retail inspection, and state-level tobacco control policies associated with T21 adoption.

**RESULTS:**

T21 adoption at the state and local level increased considerably from 1.4% of zip codes in 2015 to 40.2% in 2019. However, the T21 ordinances were disproportionally adopted in New England (82.6%) and Pacific (73.6%) regions with scarce coverage in East South Central (<0.1%), Mountain (1.6%), and West North Central regions (6.1%). The T21 policies were more likely to be adopted in areas with stronger tobacco control policies, urban areas (vs rural, adjusted odds ratio, AOR=1.25, p=0.005), areas with a larger Hispanic (AOR=1.19, p<0.0001) or Asian population (AOR=1.12, p<0.0001), and in areas where the population had higher levels of education (AOR=1.05, p<0.0001). It was less likely to be adopted in areas with larger proportions of American Indians, youths, and young adults. Nearly 40% of zip codes with tobacco retailers did not receive annual FDA tobacco retail inspections for underage sales in 2019. The average retail violation rate of underage sales of tobacco products in T21 regions was lower than in non-T21 regions.

**CONCLUSIONS:**

Disparities in T21 adoption, retail inspections, and retail compliance may limit the policy impact. Unified enforcement of youth tobacco access restrictions with resources and interventions in vulnerable communities is needed to reduce tobacco-related health disparities.

## INTRODUCTION

Youth are at the greatest risk of smoking, and smokers almost always begin experimenting with smoking cigarettes and other tobacco products before the age of 18 years^1,2^. Restricting the supply of tobacco products through age-of-sale restrictions is an important component in reducing youth smoking. Underage laws and compliance check inspections of tobacco product retailers have led to a decrease in the prevalence of youth buying cigarettes in a store^3-5^. However, social access to cigarettes has been increasing, with strong evidence indicating that youth aged <18 years can obtain their cigarettes from friends and young adults aged 18–20 years^6-8^.

In 2015, the Institute of Medicine (IOM) examined existing literature on tobacco use initiation and performed simulation studies to predict the likely health outcomes of raising the minimum legal sales age (MLSA) of tobacco products to 19, 21, or 25 years^8^. The simulation models conducted by the IOM indicate that nationwide adoption of an MLSA of 21 (T21) would result in a 25% reduction in tobacco use by those aged 15–17 years and a 12% reduction in population smoking prevalence over time. The IOM report concluded that the adoption of T21 would have a substantial positive impact on public health and would save lives^8^. Since then, there has been ongoing momentum of passing the T21 laws across the US at the state or local level. On 20 December 2019, US Congress passed the Federal T21 law to raise the minimum age of sale of tobacco products from 18 to 21 years nationwide^9,10^. Before the national law, nearly 50% of the US population had already been covered by T21, through voluntary adoption, including 540 jurisdictions across 25 different states, 16 complete states, and the District of Columbia^11^.

T21 may have relative advantages compared with other tobacco control policies (i.e. cigarette tax, smoke-free laws): 1) As policymakers are often concerned about negative economic impacts of tax and price increases on small businesses^12^, T21 may have fewer legal barriers than a cigarette tax increase; 2) According to the social construction of target population theory^13^, T21 is a policy aimed to protect youth, and thus is less politically threatening, faces fewer opposition groups, and has a shorter timeframe for adoption than smoke-free laws^12^, in addition, the T21 policy is strongly supported by a majority of youth^14^ and adults, including adult smokers^15-17^; and 3) T21 may simplify the identification of underage purchases since the color or orientation of a driver’s license changes, in many states, when a driver turns 21 years^18^.

Despite these policy advantages, the state or local adoption of T21 has been neither universal, nor was it random, and there may have been disparities in T21 policy coverage prior to the national mandate. Past studies have shown that African Americans, Hispanics/ Latinos, and less educated residents are, on average, less likely to be protected by comprehensive clean air laws in the US^19-21^. The variation in demographics and state/local jurisdictions inadvertently contributes to geographical variations in regional tobacco control laws^22^. For instance, multiple states have been actively promoting cigarette tax increases to prevent youth tobacco initiation and increase adult smoking cessation over the past two decades. Despite these efforts, there is still substantial variation in cigarette tax rates by state, ranging from $0.17/pack in Missouri to $4.50/pack in the District of Columbia in 2021^23^. Studies using small-area estimation models show considerable disparities in tobacco use, including cigarettes, smokeless tobacco, and cigars, at multilevel geographical units (e.g. county, state)^1,24^.

Rural communities are also left behind in tobacco control and tobacco prevention^25^. A cross-sectional analysis of the National Survey on Drug Use and Health from 2007 through 2014 reveals a growing disparity in cigarette smoking prevalence between rural and urban regions after taking confounding factors into account^26^. Multiple data sources also indicate a higher prevalence of smokeless tobacco and co-use with cigarettes in rural areas than urban areas^22,25,27^. Residents in rural areas are less likely to be protected by smoke-free laws and smoke-free rules in homes and cars. All these factors may contribute to a high prevalence of secondhand exposure to smoking in rural areas^28^.

Disparities in T21 adoption and implementation could introduce additional inequalities to these communities by not addressing youth access to tobacco products, which may exacerbate tobaccorelated disparities. Information on community characteristics, the policy environment, and retail inspections relative to the adoption of T21 is needed to strengthen the implementation of T21 at the state and local levels. To fill this gap, we analyzed the disparities of geographical regions, sociodemographic factors, tobacco control policies, and FDA tobacco retail inspections between T21 and non-T21 regions in 2015–2019, prior to the national T21 law. This study attempts to identify the potential gaps in T21 policy adoption and enforcement and to provide information for future initiatives by leveraging T21 laws to reduce health disparities.

## METHODS

### T21 policy data

The University of Missouri Tobacco Control Research Center compiled the T21 ordinance database^29^, which included location (county or city), State, T21 policy (yes/no), T21 enactment and effective dates, and covered population. We geocoded the T21 policy (yes/no) within each zip code from December 2015 to December 2019. We did not examine 2020 or later because T21 was passed at the national level in December 2019. This data set was linked with the following socioecological data at the zip code level.

### Census data and urbanicity

Socioeconomic status (SES) variables at the zip code level were collected from the 2014–2018 5-year American Community Survey (ACS)^30^. We selected community characteristics including the distributions (%) of age, gender, race/ethnicity, education level, owner-occupied house unit, and poverty, as all these variables are known to be associated with tobacco use prevalence and tobacco control policies^5,31^. The Rural-Urban Commuting Area (RUCA) codes from the U.S. Department of Agriculture^32^ were included to classify each zip code as urban (Metropolitan) and rural (Micropolitan, Small Town, or Rural)^5,31^. The RUCA system derives the classification based on the commuting pattern from the 2010 decennial census and 2006–2010 ACS data.

### Tobacco retailer data and FDA compliance inspection data

Tobacco retailer data were obtained from ReferenceUSA, an organization that provides business and residential data for research^33^. The commercial database from ReferenceUSA has been used in multiple tobacco retailer studies^34,35^. Using these data, we summarized the number of tobacco retailers within each zip code.

The FDA conducts two types of inspections of tobacco product retailers to determine whether a retailer is compliant with federal laws and regulations: 1) Undercover Buy (UB) inspections of youth purchase attempts, and 2) Advertising and Labeling (A&L) inspections. The UB inspections may involve the use of a minor under the supervision of the inspectors to evaluate whether a retailer sells tobacco products to individuals who are aged <18 years. The UB inspections also evaluate whether a retailer requests photo identification and verifies the date of birth for individuals aged <27 years who attempt to purchase tobacco products^36^. The FDA has maintained a database of inspection results of tobacco retailers since October 2010^36^, including details of the inspected retailers (i.e. retailer name, address, city, zip code), the decision of inspections (‘no violations observed’, ‘warning letter’, ‘civil money penalty’, ‘notobacco-sale order’), minors involved in the inspection ( yes/no/not applicable), sales to a minor (yes/no/ not applicable), and decision date. We obtained the inspection data from 1 January 2015 to 31 December 2019 and included only the UB inspections indicated as ‘minor involved’.

The inspection results were classified into two groups: 1) no violation (sale to minor = no) or violation (sale to minor = yes). We calculated the number of inspections, violations, and retail violation rates within each zip code.

### Smoking prevalence and state tobacco control policies

County-level smoking prevalence data were obtained from the County Health Rankings & Roadmaps program, which reports adult smoking rates and other health factors for each US county^37^. State cigarette tax rate data, comprehensive smoke-free laws, and tobacco retailer licensing requirements across all 50 states and the District of Columbia for each year from 2015 to 2019 were collected from the Center for Disease Control and Prevention’s State Tobacco Activities Tracking and Evaluation (STATE) System^38^. The cigarette tax data were obtained from the annual compendium on tobacco revenue and industry statistics. Comprehensive smoke-free laws include coverage in bars, restaurants, and workplaces. Tobacco retailer licensing requirement was classified as two mutually exclusive groups (yes/no).

### Statistical analysis

The data from different sources were merged at the zip code level using ArcGIS 10.5. Community characteristics, including geographical regions, tobacco control policies, adult smoking rate, SES variables, urbanicity, number of tobacco retailers, and FDA compliance inspections at the zip code level, were summarized by T21 and non-T21 areas for each year from 2015 to 2019. A multi-level logistic regression model was developed to examine social determinants associated with T21 coverage. Summary statistics for neighborhood characteristics were reported by the number of FDA tobacco retail inspections within zip codes with at least one tobacco retailer. SAS ‘Genmod’ procedure^39^ was used to account for the spatial autocorrelation among geographical units, as neighboring geographical units might be more similar than distant ones. Adjusted odds ratios (AOR) along with associated 95% confidence intervals (CI) from the multi-level analysis are reported. Statistical analyses were performed using SAS 9.4 (Cary, NC) and a p<0.05 was considered statistically significant.

## RESULTS

T21 coverage increased dramatically from 1.4% to 40.2% of total zip codes from December 2015 to December 2019 (Table 1), with a higher percentage of T21 adoption in urban areas compared to rural areas. For instance, from December 2019, 50.0% of zip codes in urban areas adopted T21 compared to only 31.4% in rural areas. T21 was more prevalent in Northeastern zip codes than Western, Southern, or Midwestern zip codes. The New England region had the highest coverage (82.6%) in December 2019, followed by the Pacific region (73.6%), while the West North Central (6.1%), Mountain (1.6%), and East South Central (<0.1%) regions had the lowest coverage. T21 areas also had higher statelevel cigarette taxes and were more likely to adopt tobacco licensing requirements and comprehensive smoke-free laws than non-T21 areas. As of December 2019, only 19.8% of zip codes in states with no license requirements adopted T21, in comparison with 46.5% of zip codes with tobacco retail licensing covering e-cigarettes or other tobacco products; 24.9% of zip codes in states with no comprehensive smoke-free laws adopted T21 versus 54.2% of zip codes in states with comprehensive smoke-free laws.

**Table 1:** Geographical differences between T21 and Non-T21 regions, 2015–2019

	*2015*		*2016*		*2017*		*2018*		*2019*	
	*Non-T21*	*T21*	*Non-T21*	*T21*	*Non-T21*	*T21*	*Non-T21*	*T21*	*Non-T21*	*T21*
**Number of zip codes,** n (%)	32522 (98.6)	450 (1.4)	30297 (91.9)	2675 (8.1)	29427 (89.2)	3545 (10.8)	27832 (84.4)	5140 (15.6)	19710 (59.8)	13262 (40.2)
**Urbanicity^a^**, (%)										
Urban	97.6	2.4	87.3	12.7	82.3	17.7	76.2	23.8	50.0	50.0
Rural	99.6	0.4	96.1	3.9	95.6	4.4	91.9	8.1	68.6	31.4
**Census,** (%)										
Midwest	99.3	0.7	97.4	2.6	96.1	3.9	94.6	5.4	69.9	30.1
Northeast	95.0	5.0	91.6	8.4	79.8	20.2	63.4	36.6	35.1	64.9
South	100.0	0.0	99.5	0.5	99.5	0.5	98.9	1.1	64.3	35.7
West	98.6	1.4	67.3	32.7	66.7	33.3	59.9	40.1	59.0	41.0
**Census region,** (%)										
East North Central	100.0	0.1	98.4	1.6	97.2	2.8	94.9	5.1	54.0	46.0
East South Central	100.0	0.0	100.0	0.0	100.0	0.0	100.0	0.0	100.0	0.0
Middle Atlantic	95.2	4.8	92.2	7.8	75.9	24.1	70.8	29.2	42.9	57.1
Mountain	100.0	0.0	100.0	0.0	99.9	0.1	99.7	0.3	98.4	1.6
New England	94.5	5.5	90.1	9.9	88.9	11.1	46.5	53.5	17.4	82.6
Pacific	97.5	2.5	40.3	59.7	39.2	60.8	26.8	73.2	26.4	73.6
South Atlantic	100.0	0.0	99.0	1.0	99.0	1.0	99.0	1.0	70.8	29.2
West North Central	98.3	1.7	95.9	4.1	94.6	5.4	94.3	5.7	93.9	6.1
West South Central	100.0	0.0	100.0	0.0	100.0	0.0	98.1	1.9	31.5	68.5
**Tobacco retail licensing^b^,** ( % )										
No	99.4	0.6	98.3	1.7	97.2	2.8	92.2	7.8	80.2	19.8
Yes	98.3	1.7	89.9	10.1	86.8	13.2	82.0	18.0	53.5	46.5
**Comprehensive smoke-free air laws^c^,** (%)										
No	99.4	0.6	99.1	0.9	99.1	0.9	98.5	1.5	75.1	24.9
Yes	97.8	2.2	85.3	14.7	80.3	19.7	71.6	28.4	45.8	54.2
State cigarette tax^d^, ($) mean (SD)	1.6 (1.1)	3.0 (1.5)	1.6 (1.1)	1.6 (1.3)	1.6 (1.1)	2.9 (0.9)	1.7 (1.1)	2.8 (1.0)	1.4 (0.9)	2.4 (1.2)

a The Rural-Urban Commuting Area (RUCA) codes are from the USDA. A binary indicator was created for urban (Metropolitan) versus rural (Micropolitan, Small Town and Rural).

b State tobacco licensing requirements were collected from the CDC’s State Tobacco Activities Tracking and Evaluation (STATE) System.

c Comprehensive smoke-free laws were collected from the CDC’s State Tobacco Activities Tracking and Evaluation (STATE) System. Comprehensive smoke-free laws include coverage in bars, restaurants, and workplaces.

d State cigarette tax rate data for each year were collected from the CDC’s State Tobacco Activities Tracking and Evaluation (STATE) System.

Table 2 presents the comparison of sociodemographic characteristics between T21 and non-T21 regions from 2015 to 2019. From December 2019, in comparison with non-T21 regions, T21 regions had more tobacco retailers (12.8 vs 9.5), lower adult smoking prevalence (15.6% vs 17.8%), lower retail violation rates of underage sales (11.6% vs 14.0%), larger populations (12207 vs 7843), smaller proportions of non-Hispanic Whites (73.6% vs 80.8%), higher proportions of Hispanics (12.8% vs 6.1%) and Asians (3.5% vs 1.1%), and higher proportions of college graduates (22.6% vs 16.8%). From 2015 to 2019, the gap in disparities of T21 adoption narrowed. For instance, the gap in the proportion of college graduates between T21 regions and non-T21 regions decreased from 16.8% in 2015 to 5.8% in 2019.

**Table 2:** Zip code level characteristics between T21 and Non-T21 regions, 2015–2019 ^a^

	*2015*		*2016*		*2017*		*2018*		*2019*	
	*Non-T21*	*T21*	*Non-T21*	*T21*	*Non-T21*	*T21*	*Non-T21*	*T21*	*Non-T21*	*T21*
**Number of zip codes,** n (%)	32522 (98.6)	450 (1.4)	30297 (91.9)	2675 (8.1)	29427 (89.2)	3545 (10.8)	27832 (84.4)	5140 (15.6)	19710 (59.8)	13262 (40.2)
**Number of tobacco retailers^b^**	10.7	18.4	10.1	19.4	9.9	18.6	9.9	16.0	9.5	12.8
**Adult smoking rate^c^,** (%)	16.9	15.1	17.2	13.2	17.3	13.6	17.4	14.1	17.8	15.6
**Previous year inspection^d^,** (%)										
Number of inspections	4.1	8.2	3.9	3.8	3.9	4.1	4.2	5.0	4.3	4.4
Number of violations	0.6	0.8	0.5	0.4	0.6	0.5	0.6	0.4	0.6	0.5
Retail violation of underage sales	14.7	9.8	12.9	9.3	14.8	11.9	13.1	8.3	14.0	11.6
**SES variables^e^**										
**Population,** n	**9498**	**16844**	**8633**	**20539**	**8480**	**18887**	**8386**	**16167**	**7843**	**12207**
**Gender,** (%)										
Male	50.2	49.2	50.2	50.1	50.2	50.0	50.3	49.9	50.4	50.0
**Race,** (%)										
White	78.1	64.6	79.8	56.5	80.0	60.5	79.9	67.2	80.8	73.6
African American	7.5	7.8	7.6	6.8	7.5	7.3	7.7	6.2	7.6	7.4
Hispanic/Latino	8.7	16.0	7.5	23.2	7.4	20.2	7.4	16.5	6.1	12.8
Asian	2.0	7.0	1.5	8.6	1.3	7.8	1.3	6.2	1.1	3.5
American Indian	1.7	0.3	1.7	0.8	1.8	0.7	1.8	0.7	2.4	0.5
**Age** (years)										
15-17	3.9	3.5	3.9	3.6	3.9	3.7	3.9	3.7	3.9	3.8
18-20	4.1	4.7	4.0	4.6	4.0	4.5	4.0	4.3	3.9	4.2
21-24	4.8	5.5	4.8	5.7	4.8	5.4	4.8	5.2	4.7	5.0
**Education level** (aged >25 years), (%)										
College +	18.9	35.7	18.4	27.9	18.0	28.6	17.6	27.7	16.8	22.6
**Other,** (%)										
Owner occupied housing unit	59.8	51.4	60.5	49.7	60.5	52.9	60.7	54.3	60.4	58.6
Poverty	14.8	12.6	14.7	15.3	14.8	14.0	14.9	13.7	15.5	13.7

a The mean of each attribute at the zip code level was reported in T21 and non-T21 regions each year.

b Tobacco retail data were obtained from ReferenceUSA and were identified as all businesses that likely sold tobacco products as of December 2016 using North American Industry Classification System codes, such as 445110 (supermarkets and grocery stores), 445120 (convenience stores or food marts), 445310 (beer, wine and liquor stores), 446110 (pharmacy and drug stores), 447110 (gasoline stations with convenience stores), 447190 (other gasoline stations), 452990 (discount stores and general stores), 452910 (warehouse clubs and superstores), and 453991 (tobacco stores). We further excluded national chain retailers that do not sell tobacco products (e.g. Target, CVS, Whole Foods, Dollar Tree) and removed duplicate records.

c County-level smoking prevalence data were obtained from the County Health Rankings & Roadmaps program.

d The underage tobacco inspection data were obtained from the FDA compliance inspection database. Retail violation of underage sales included warning letter, civil money penalty, and no-tobacco-sale order.

e SES variables were obtained from the 2014–2018 American Community Survey data.

Table 3 depicts the multi-level analysis of factors associated with T21 adoption in December 2019. T21 ordinances were more likely to be adopted in areas with comprehensive smoke-free laws (AOR=3.63; 95% CI: 2.74–4.82, p<0.0001), tobacco licensing requirements (AOR=1.96; 95% CI: 1.42–2.71, p<0.0001), and in urban (vs rural) areas (AOR=1.25; 95% CI: 1.07–1.46, p=0.005), but were less likely to be adopted in zip codes with higher retail violations of underage sales (AOR=0.72; 95% CI: 0.61–0.85, p<0.0001). We observed a higher likelihood of T21 adoption among zip codes with higher proportions of Hispanics/Latinos (AOR=1.19; 95% CI: 1.14–1.24, p<0.0001), Asians (AOR=1.12; 95% CI: 1.06–1.19, p<0.0001), and college graduates (AOR=1.05; 95% CI: 1.02–1.08, p<0.0001). The likelihood of T21 adoption was lower in zip codes with higher proportions of American Indians (AOR=0.87; 95% CI: 0.84–0.90, p<0.0001), individuals aged 15–17 years (AOR=0.89; 95% CI: 0.83–0.96, p<0.0001) and individuals aged 18–20 years (AOR=0.97; 95% CI: 0.94–1.00, p<0.0001).

**Table 3:** Multi-level analysis of factors in association with voluntary T21 adoption as of December 2019

*Predictive variables*	*AOR (95% CI)*	*p*
**State tobacco regulation**		
Comprehensive smoke-free air laws (yes vs no)	3.63 (2.74–4.82)	<0.0001
Tobacco licensing (yes vs no)	1.96 (1.42–2.71)	<0.0001
**Local characteristics**		
Urban vs rural^a^	1.25 (1.07–1.46)	0.005
Retail violation of minor sales^b^	0.72 (0.61–0.85)	<0.0001
**Zip code level SES^c^**(%)		
Non-Hispanic Black	1.03 (1.00–1.06)	0.0759
Hispanic	1.19 (1.14–1.24)	<0.0001
Asian	1.12 (1.06–1.19)	<0.0001
American Indians	0.87 (0.84–0.90)	<0.0001
15–17 years old	0.89 (0.83–0.96)	<0.0001
18–20 years old	0.97 (0.94–1.00)	<0.0001
Bachelor’s degree and above	1.05 (1.02–1.08)	0.003
Persons living in poverty	1.01 (0.98–1.03)	0.0553

a The Rural-Urban Commuting Area (RUCA) codes are from the USDA. A binary indicator was created for urban (Metropolitan) versus rural (Micropolitan, Small Town and Rural).

b The underage tobacco inspection data were obtained from the FDA compliance inspection database. Retail violation of underage sales included warning letter, civil money penalty, and no-tobacco-sale order.

c Per 10% increase. SES: socioeconomic status. AOR: adjusted odds ratio.

Table 4 presents the average number of FDA tobacco retail inspections within each US zip code in 2019. Of 26150 zip codes with tobacco retailers, 38.4% (10048) had no FDA compliance inspections involving minors in 2019, 11.5% (3001) had 1 compliance inspection, 14.1% (3690) had 2–3 compliance inspections, 17.1% (4481) had 4–9 compliance inspections, and 18.9% (4930) had ≥10 compliance inspections. Zip codes with a higher number of compliance inspections were more likely to be located in urban areas than rural areas. These zip codes also had a higher number of tobacco retailers and a larger population. For instance, 74.4% of zip codes with ≥10 compliance inspections in 2019 were located in urban areas compared to 57.6% of zip codes with 4–9 inspections, 48.1% with 2–3 inspections, 44.2% with 1 inspection, and 46.2% with zero inspections. Overall, zip codes with a higher (vs lower) number of compliance inspections had higher proportions of African Americans and Hispanics, adults aged 18–44 years, and people with a Bachelor’s degree or higher.

**Table 4:** FDA tobacco retailer compliance inspections of sales to minors in each zip code, 2019 (N=143762)

	*Number of compliance inspections in each zip code^a^*					
	*0*	*1*	*2-3*	*4-9*	*>10*	*Total*
**Zip codes,** n (%)	10048 (38.4)	3001 (11.5)	3690 (14.1)	4481 (17.1)	4930 (18.9)	26150 (100)
**Zip code level characteristics**						
Number of tobacco retailers	10.1	8.9	10.6	18.2	37.5	15.6
Number of inspections	0.0	1.0	2.4	6.0	22.8	5.0
Number of violations	0.0	0.1	0.4	0.9	3.3	0.7
**Population,** n	**8789**	**6999**	**8204**	**13661**	**26731**	**12197**
**Urban,** (%)	**46.1**	**44.2**	**48.1**	**57.6**	**74.4**	**53.5**
**Sex,** (%)						
Male	50.3	50.3	50.1	49.5	49.0	49.9
**Race/ethnicity,** (%)						
Caucasian	75.8	82.7	82.7	77.1	65.0	75.7
African American	6.5	6.2	6.5	9.4	14.6	8.5
Hispanic	10.7	6.7	6.3	8.4	13.7	9.8
Asian	2.3	1.9	1.7	2.2	3.6	2.4
American Indian	2.5	0.9	0.9	0.8	0.6	1.4
**Age** (years), (%)						
10-17	10.2	10.4	10.4	10.5	10.1	10.3
18-29	14.1	13.4	13.5	14.6	17.1	14.6
30-44	17.2	17.6	17.6	18.2	19.2	17.9
45-64	29.1	29.8	29.5	28.2	26.3	28.5
**Socioeconomic status,** (%)						
Bachelor's degree or higher	18.7	18.2	18.9	21.3	23.5	20.1
Poverty	14.8	13.7	13.7	14.4	16.6	14.8
**County-level characteristics,** (%)						
Adult smoking prevalence	16.6	17.2	17.3	17.1	16.6	16.9

a Zip codes were divided into 5 groups based on the number of tobacco retail inspections involving minors.

## DISCUSSION

This study evaluated multiple factors associated with T21 adoption in the US from 2015 to 2019, before the nationwide adoption of T21, to identify disparities between regions that adopted the policy and those that did not. We found significant inequalities in the coverage of state/local T21 by geographical distribution, rurality, demographics, SES factors, and tobacco control policies. These findings add to the growing literature^40,41^ that state or local T21 policies were not uniformly adopted in the US. We further used the proxy measure of FDA retail inspections to inform potentially differential enforcement of T21 laws by region and other ecological factors. Before the Federal T21 mandate, a patchwork of different MLSAs and varying ordinances with varying enforcement actions by states and localities likely led to jurisdiction and implementation variations that limited the impact of T21, and therefore likely limited the potential impact on reducing tobacco-related health disparities.

We found a significant disparity in the adoption of state or local T21 by geographical distribution. As of December 2019, T21 was more likely to be adopted in the Northeast (64.9%) and West regions (41.0%) than in the South (35.7%) and Midwest (30.1%). It is promising to observe the narrowing racial disparity between T21 and non-T21 regions with high coverage for some racial/ethnic minority populations, including Hispanics/Latinos and Asians. However, the T21 policy coverage in areas with high proportions of American Indians persistently fell behind other racial/ethnic groups from 2015 to 2019. American Indians have a high prevalence of tobacco use and early initiation of tobacco in childhood^22^.

More resources, culturally tailored programming, and policy enactments are needed to help reduce tobacco use in this vulnerable subpopulation.

Besides differential coverage, provisions within these local, state, and territorial T21 policies vary widely in terms of tobacco definition, age verification, signage, enforcement, education level, and flavor bans^42,43^. For instance, the tobacco retail licensing fee ranges from $20 in Hawaii and Utah to $265 in California. Appearance age for ID verification ranges from 21 to 30 years old, and multiple state tobacco ordinances do not specify an appearance age. Variation in the implementation and enforcement of T21 policy across regions could undermine the potential impact of this policy.

We found the adult smoking prevalence was consistently lower in T21 regions than non-T21 regions from 2015 to 2019. The prevalence of cigarette smoking among US adults aged ≥18 years was highest among people living in the Midwest (16.4%) and the South (15.4%), and lowest among those living in the Northeast (12.8%) and West (10.4%) U.S. Census regions^44^. However, state/local adoption of T21 was inversely associated with smoking prevalence. There were significant disparities in adoption within the West region between the Pacific (73.6%) and Mountain (1.6%) regions. Some of the most significant disparities in health outcomes due to tobacco, such as morbidity and mortality related to lung cancer and coronary heart disease, were also found in regions with high numbers of adult smokers^45,46^.

This study adds to the literature in that the T21 policy was less likely to be adopted in rural areas than urban areas, as previous studies have suggested for other tobacco-related policies^47^. We also found zip codes in rural areas had fewer FDA tobacco retail inspections, with a majority of zip codes with no inspections located in rural areas in contrast to 74.4% of zip codes with ≥10 inspections located in urban areas. Rural communities in the US, characterized by low income, high unemployment, and low education level, have a higher prevalence of tobacco use and tobacco-related complications than urban communities^48^. These urban-rural gaps may be attributable to limited tobacco control resources and tobacco intervention programming in rural regions. Furthermore, rural areas are often less politically progressive than large cities^49^. Due to the lack of financial and policy advocacy support, policymakers in rural areas face challenges to aggressively enact more robust tobacco control policies, which could lead to tobacco industry legal action. Strong federal policies, increasing funding for tobacco prevention, and targeted community-based interventions may help strengthen tobacco control and regulatory benefits to vulnerable populations among rural Americans. A recent state-wide large-scale survey of middle and high school students from 6th to 12th grade^50^ found an increase in the prevalence of current e-cigarette use from 2018 to 2019, with a larger increase in rural areas than in urban areas. Remarkably, the increase was smaller in T21 than in non-T21 regions, suggesting that T21 policies may help mitigate these increases in youth e-cigarette use^50^. Due to the weak adoption of T21 in rural areas, it is critical to effectively enforce this new tobacco control ordinance in rural areas.

This study also found college graduate rates to be a significant predictor for T21 adoption, with the proportion of college graduates higher in T21 regions than in non-T21 regions. College graduates may be more aware of risks associated with tobacco use and more compliant with tobacco regulation compared to people with lower education levels. Increased knowledge of tobacco control policies may also help change social norms and accelerate support and compliance of policy across populations^51,52^. Dai et al.^53^ found that knowledge of the MLSA was inversely associated with the intention to use tobacco among youth. Therefore, social media campaigns and public education to raise awareness of T21 are warranted, especially in disadvantaged areas.

The national passage of the T21 law has opened a new era in tobacco control that could further reduce tobacco-related health disparities. Effective implementation and enforcement of the national T21 law is critical in diffusing the policy effects. Although T21 is now mandated nationwide in the US, we speculate similar gaps might exist in the enforcement of T21 as regions with weak tobacco control and high tobacco use prevalence might lack the resources to enforce T21 effectively. Given the inverse relationship between education level and tobacco use, disparities in the implementation of T21 may leave a gap in the protection of those already most vulnerable to tobacco-related diseases. Continued efforts to increase awareness and support of T21 in the general public and implement multifaceted strategies to ensure T21 compliance are needed to optimize the effectiveness of the policy.

### Limitations

This study has some limitations. First, there might be other confounding factors affecting the adoption of T21. However, we have selected multi-level risk factors that have previously been shown to be associated with tobacco control policy and tobacco use behaviors in literature and included time-varying variables in this five-year longitudinal study. Second, we coded the T21 enforcement as yes versus no at each zip code and did not consider the strength of local/state T21 policies, which can widely differ across states^40^. Third, we used FDA tobacco retail inspections as a proxy measure, which did not include other retail inspections conducted at state and local levels.

## CONCLUSIONS

This study highlights geographical variation and socioecological factors associated with disparities in T21 adoption, retail inspections, and retail compliance of underage sales laws. The empirical evidence supports initiatives to strengthen enforcement of age restriction in tobacco sales, education of tobacco retailers, and surveillance of new avenues of access such as online sales that may increase in popularity due to the barriers set forth from the national enactment of the T21 law. A comprehensive approach integrating the T21 law with other tobaccorelated policies and evidence-based interventions can help reduce tobacco initiation among adolescents^54^. Consistent implementation and enforcement of T21 could serve as an effective tool to ameliorate tobaccorelated disparities.

## Data Availability

The data supporting this research are available from the authors on reasonable request.
