# Supplementary material for: Geographical distribution and social determinants of Tobacco 21 policy adoption and retail inspections in the United States, 2015–2019
Source: Tob Induc Dis. 2021 Sep 16;19:55. doi: 10.18332/tid/140148 (PMC8445336; doi:10.18332/tid/140148)
Supplement: Supplementary file 2 [file TID-19-71-s2.pdf]

Supplemental File 1: Design of Aims 2 and 3

Experimental (8 x 8 balanced lattice) design in which each participant is randomized to one of nine repetitions (depicted as columns here) and completes a discrete choice experiment while viewing eight stimuli (packs) within each of eight blocks presented in random order. Thus, each participant was asked to complete eight discrete choice tasks.

See Cochran and Cox Plan 10.5 for the full design.[1]

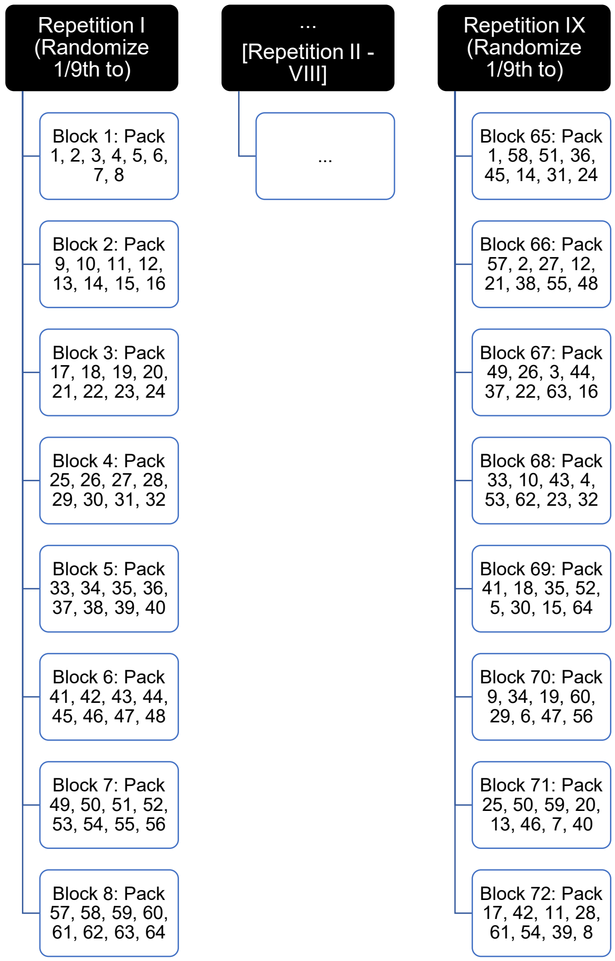

1. Cochran WD, Cox GM. *Experimental designs.* 2 ed. New York: John WIley & Sons; 1992.

Supplemental Table 1. Pairwise comparisons for Study 1

**Shape Comparisons**	**Diff**	**SE(diff)**	**Z-value**	**p-value**
**1 vs 2**	-1.1319	0.114657	-9.87204	<0.001
**1 vs 3**	-1.1921	0.11705	-10.1846	<0.001
**1 vs 4**	-1.3392	0.11924	-11.2311	<0.001
2 vs 3	-0.0602	0.093385	-0.64464	0.52
**2 vs 4**	-0.2073	0.096117	-2.15675	0.03
3 vs 4	-0.1471	0.098959	-1.48648	0.14
**Color Saturation Comparison**	**Diff**	**SE(diff)**	**Z-value**	**p-value**
**1 vs 2**	-0.759	0.095936	-7.9115	<0.001
**1 vs 3**	-0.7991	0.097351	-8.20841	<0.001
**1 vs 4**	-0.73	0.103161	-7.07634	<0.001
2 vs 3	0.0291	0.054456	0.534374	0.59
2 vs 4	-0.011	0.054041	-0.20355	0.84
3 vs 4	-0.0401	0.053035	-0.75611	0.45
**Logo Size Comparison**	**Diff**	**SE(diff)**	**Z-value**	**p-value**
1 vs 2	0.0542	0.068473	0.791557	0.43
**1 vs 3**	-0.228	0.06618	-3.44515	<0.001
1 vs 4	-0.1126	0.069164	-1.62801	0.10
2 vs 3	-0.2822	0.06425	0.534374	0.59
2 vs 4	-0.1668	0.06732	-0.20355	0.84
3 vs 4	-0.0401	0.064987	-0.75611	0.45

Supplemental Table 2. Pairwise comparisons for Study 2's Appeal Task

Design Comparison	Diff	SE(diff)	Z-value	p-value
1 vs 2	0.1040	0.09631	1.079851	0.28
**1 vs 3**	0.3669	0.103809	3.534385	<0.01
**1 vs 4**	0.9000	0.116715	7.71106	<0.01
2 vs 3	-0.0602	0.054874	-1.09705	0.27
**2 vs 4**	-0.2073	0.053835	-3.85063	<0.01
**3 vs 4**	-0.1471	0.053256	-2.76215	0.01
**Color Comparison**	**Diff**	**SE(diff)**	**Z-value**	**p-value**
**1 vs 2**	1.1636	0.113066	10.2913	<0.01
**1 vs 3**	1.0136	0.108706	9.324246	<0.01
**1 vs 4**	0.7702	0.104479	7.371824	<0.01
**1 vs 5**	0.5645	0.109933	5.134968	<0.01
**1 vs 6**	0.8826	0.108706	8.119159	<0.01
**1 vs 7**	0.6542	0.109864	5.954637	<0.01
**1 vs 8**	0.6689	0.08	8.36125	<0.01
**2 vs 3**	0.7111	0.086964	8.176907	<0.01
**2 vs 4**	0.4677	0.086964	5.378062	<0.01
**2 vs 5**	0.2620	0.08	3.275	<0.01
**2 vs 6**	0.5801	0.08	7.25125	<0.01
**2 vs 7**	0.3517	0.08	4.39625	<0.01
**2 vs 8**	0.3664	0.08	4.58	<0.01
**3 vs 4**	-0.3934	0.104402	-3.76811	<0.01
**3 vs 5**	-0.5991	0.10986	-5.45332	<0.01
**3 vs 6**	-0.281	0.108632	-2.58671	0.01
**3 vs 7**	-0.5094	0.109791	-4.63972	<0.01
**3 vs 8**	-0.4947	0.0799	-6.19149	<0.01
**4 vs 5**	-0.4491	0.105367	-4.26226	<0.01
4 vs 6	-0.1310	0.104086	-1.25857	0.21
**4 vs 7**	-0.3594	0.105295	-3.41327	<0.01
**4 vs 8**	-0.3447	0.0736	-4.68342	<0.01
5 vs 6	0.1124	0.099663	1.127796	0.26
5 vs 7	-0.1160	0.100925	-1.14936	0.25
5 vs 8	-0.1013	0.0672	-1.50744	0.13
6 vs 7	0.0897	0.106561	0.841771	0.40
6 vs 8	0.1044	0.0754	1.384615	0.17
**7 vs 8**	-0.2137	0.082752	-2.5824	0.01

Supplemental Table 3. Pairwise comparisons for Study 2's Least Harm Task

Design Comparison	Diff	SE(diff)	Z-value	p-value
1 vs 2	-0.1051	0.113208	-0.92838	0.35
**1 vs 3**	-1.0699	0.108220	-9.88631	<0.01
1 vs 4	0.1349	0.125067	1.078623	0.28
2 vs 3	-0.0602	0.054874	-1.09705	0.27
**2 vs 4**	-0.2073	0.053835	-3.85063	<0.01
**3 vs 4**	-0.1471	0.053256	-2.76215	<0.01
**Color Comparison**	**Diff**	**SE(diff)**	**Z-value**	**p-value**
**1 vs 2**	0.5054	0.103726	4.872451	<0.01
**1 vs 3**	0.7694	0.100364	7.666131	<0.01
**1 vs 4**	0.8964	0.095390	9.397213	<0.00
**1 vs 5**	0.9375	0.099702	9.403039	<0.01
**1 vs 6**	0.5536	0.092825	5.963938	<0.01
**1 vs 7**	0.7278	0.093994	7.743057	<0.01
**1 vs 8**	0.4141	0.100069	4.138146	<0.01
**2 vs 3**	0.2640	0.06780	3.893805	<0.01
**2 vs 4**	0.3910	0.076671	5.099695	<0.01
**2 vs 5**	0.4321	0.076671	5.635750	<0.01
2 vs 6	0.0482	0.06780	0.710914	0.48
**2 vs 7**	0.2224	0.06780	3.280236	<0.01
2 vs 8	-0.0913	0.06780	-1.346610	0.18
3 vs 4	0.1270	0.099892	1.271373	0.20
3 vs 5	0.1681	0.104017	1.616076	0.11
**3 vs 6**	-0.2158	0.097445	-2.214580	0.03
3 vs 7	-0.0416	0.098560	-0.422080	0.67
**3 vs 8**	-0.3553	0.104369	-3.40426	<0.01
4 vs 5	0.0411	0.099227	0.414201	0.68
**4 vs 6**	-0.3428	0.092315	-3.71339	<0.01
4 vs 7	-0.1686	0.093490	-1.80340	0.07
**4 vs 8**	-0.4823	0.099596	-4.84256	<0.01
**5 vs 6**	-0.3839	0.096763	-3.96741	<0.01
**5 vs 7**	-0.2097	0.097886	-2.14229	0.03
**5 vs 8**	-0.5234	0.103733	-5.04564	<0.01
6 vs 7	0.1742	0.090871	1.91700	0.06
6 vs 8	-0.1395	0.097142	-1.43605	0.15
**7 vs 8**	-0.3137	0.072809	-4.30851	<0.01

Supplemental Table 4. Pairwise comparisons for Study 2's Personal Style Task

**Design Comparison**	**Diff**	**SE(diff)**	**Z-value**	**p-value**
1 vs 2	-0.0353	0.098502	-0.35837	0.72
**1 vs 3**	0.3062	0.106142	2.884818	<0.01
**1 vs 4**	0.8674	0.119112	7.282214	<0.01
2 vs 3	-0.0602	0.054874	-1.09705	0.27
**2 vs 4**	-0.2073	0.053835	-3.85063	<0.01
**3 vs 4**	-0.1471	0.053256	-2.76215	0.01

**Color Comparison**	**Diff**	**SE(diff)**	**Z-value**	**p-value**
**1 vs 2**	0.5669	0.117631	4.819293	<0.01
**1 vs 3**	1.3016	0.112359	11.58427	<0.01
**1 vs 4**	1.0202	0.108747	9.381416	<0.01
**1 vs 5**	0.8465	0.10371	8.162166	<0.01
**1 vs 6**	0.6593	0.10827	6.089378	<0.01
**1 vs 7**	0.7974	0.11019	7.23656	<0.01
**1 vs 8**	0.7682	0.110885	6.927882	<0.01
**2 vs 3**	0.7347	0.0795	9.241509	<0.01
**2 vs 4**	0.4533	0.086703	5.228193	<0.01
**2 vs 5**	0.2796	0.086703	3.224802	<0.01
2 vs 6	0.0924	0.0795	1.162264	0.25
**2 vs 7**	0.2305	0.0795	2.899371	<0.01
**2 vs 8**	0.2013	0.0795	2.532075	0.01
**3 vs 4**	-0.2814	0.108674	-2.5894	0.01
**3 vs 5**	-0.4551	0.103634	-4.39143	<0.01
**3 vs 6**	-0.6423	0.108197	-5.93639	<0.01
**3 vs 7**	-0.5042	0.110118	-4.57871	<0.01
**3 vs 8**	-0.5334	0.110814	-4.81349	<0.01
4 vs 5	-0.1737	0.099706	-1.74213	0.08
**4 vs 6**	-0.3609	0.104441	-3.45554	<0.01
**4 vs 7**	-0.2228	0.10643	-2.0934	0.04
**4 vs 8**	-0.2520	0.107149	-2.35186	0.02
5 vs 6	-0.1872	0.099186	-1.88737	0.06
5 vs 7	-0.0491	0.101278	-0.4848	0.63
5 vs 8	-0.0783	0.102034	-0.76739	0.44
6 vs 7	0.1381	0.105943	1.30353	0.19
6 vs 8	0.1089	0.106666	1.020948	0.31
7 vs 8	-0.0292	0.081237	-0.35944	0.72

© 2021 Lee J.G.L. et al.
