# Supplementary material for: Geographical distribution and social determinants of Tobacco 21 policy adoption and retail inspections in the United States, 2015–2019
Source: Tob Induc Dis. 2021 Sep 16;19:55. doi: 10.18332/tid/140148 (PMC8445336; doi:10.18332/tid/140148)
Supplement: Supplementary file 3 [file TID-19-71-s3.pdf]

Tobacco Induced Diseases

Research Paper

Changes to cigarette packaging influence US consumers' choices: Results of two discrete-choice experiments to inform regulation

Joseph G. L. Lee[1,2], Kevin F. O'Brien[3], Tiffany M. Blanchflower[4], Gunnar Swanson[5], Paige E. Averett[6], Kyle R. Gregory[1]

ABSTRACT

INTRODUCTION While plain packaging of tobacco products has emerged as a policy intervention to reduce smoking, regulators in the US have limited ability to implement plain packaging. We sought to identify the impact of subtle changes to cigarette packaging (Study 1) and how packaging design influenced participant choices based on appeal, harm, and style (Study 2).

METHODS We conducted two discrete-choice experiments with US adult smokers online in 2018. In Study 1 (n=285), we assessed participants' selections based on subtle changes to pack design features (dimensions, color saturation, logo size). In Study 2 (n=284), we assessed three choices in which participants selected packs based on appeal, harmfulness, and best match to their personal style. Study 2 packs varied by color hue, design with different levels of organic labeling and natural imagery, and color saturation.

RESULTS Pack designs influenced smokers' choices. In Study 1, pack dimensions and color saturation emerged as the most important features, and, in Study 2, design and color hue were the most influential characteristics.

CONCLUSIONS Regulators should consider how the design of cigarette packages may influence consumers' perceptions and choices.

AFFILIATION
1 Department of Health Education and Promotion, College of Health and Human Performance, East Carolina University, Greenville, United States
2 Cancer Prevention and Control Program, UNC Lineberger Comprehensive Cancer Center, Chapel Hill, United States
3 Department of Biostatistics, College of Allied Health Sciences, East Carolina University, Greenville, United States
4 Department of Interior Design and Merchandising, College of Health and Human Performance, East Carolina University, Greenville, United States
5 College of Fine Arts and Communication, East Carolina University, Greenville, United States
6 School of Social Work, College of Humanities and Social Sciences, North Carolina State University, Raleigh, United States

CORRESPONDENCE TO
Joseph G.L. Lee. Department of Health Education and Promotion, College of Health and Human Performance, East Carolina University, 1000 E. 5th St. Stop 529, Greenville, NC 27858, United States.
E-mail: leejose14@ecu.edu
ORCID ID: https://orcid.org/0000-0001-9698-649X

KEYWORDS
tobacco products, United States Food and Drug Administration, product packaging, marketing

Received: 21 May 2021
Revised: 10 July 2021
Accepted: 11 July 2021

Tob. Induc. Dis. 2021;19(September):70 https://doi.org/10.18332/tid/140137

Published by European Publishing. © 2021 Lee J.G.L. et al. This is an Open Access article distributed under the terms of the Creative Commons Attribution 4.0 International License. (https://creativecommons.org/licenses/by/4.0/)

INTRODUCTION

Tobacco products were historically less regulated than strawberry jam in the US[1] despite being a, if not the, leading preventable cause of early death and disability[2]. Marketing literature suggests that when a brand introduces a new product to market it should be reminiscent of existing brand packaging, drawing on design elements from its brand line; in doing so, it signals to the consumer that the new product aligns with the existing branding[3-5]. More specifically, this research suggests that a new product packaging should: 1) feature unique packaging characteristics, 2) align with existing brand strengths, and 3) be noticeably different to consumers[3-5]. The last function is vital, as a consumer's ability or inability to notice newness can trigger a change in product purchasing patterns and perceptions[3-6]. Particular to perceptions of tobacco products, previous research has shown that changes to product packaging can increase consumers' perception of healthfulness and harmfulness, even in instances when the physical product (e.g. the actual cigarette) has not changed[7].

Changes to cigarette packaging, like other consumable goods, sway consumers' purchasing behaviors and product perceptions[8-10]. Substantial evidence indicates that cigarette pack design matters to consumer perceptions of product harm[7,11]. Internal tobacco industry research revealed that cigarette product packaging changes are designed to influence smokers' purchasing behaviors, crafting products that are more appealing to specific demographic and identity groups[12]. For instance, changes in smoking by women have been shown to be related to a carefully calibrated approach to create packages with 'feminine' design features[13]. Packaging color has been identified as a particularly important characteristic in marketing cigarettes[7,14] and natural imagery represents a strategy that has been used to give a healthy 'halo' effect to products[15]. Supporting this research further, there has been a myriad of studies that have provided compelling evidence that plain packaging with its absence of brand features influences consumer behaviors and perceptions[16-21], although plain packaging is likely not a viable policy option in the US given constitutional protections for marketing as corporate speech[22].

Until 2009, there was no meaningful regulation of packaging in the US, and thus there was a limited need for research on the impacts of changes to cigarette packaging[23]. Globally, a substantial focus has been on the evidence for plain packaging[16]. While there is substantial literature on product packaging and the visual design of products in the fields of marketing and consumer behavior, in the US tobacco regulatory science research still has gaps that can hinder the ability of the U.S. Food and Drug Administration (FDA) to create and defend science-based regulations[24]. First, tobacco industry document research and observational studies[7,11] offer courts evidence but with less weight than experimental research. Second, much of the scientific literature on cigarette product labeling changes was conducted outside of the US, often where plain packaging and large graphic warning labels are the design standard enforced by regulating bodies[18,25]. Third, research has not focused on small changes to cigarette packaging, leaving gaps in knowledge regarding how substantive a change would be required to indicate a new product. Combined, these limitations to the literature impede the development and defence of science-based regulations even when there is a strong scientific consensus that design changes to cigarette packs substantially impact population health.

A theoretical framework, the Context of Consumption Framework[26], supports the importance of understanding the role of visual product design in cigarette packaging. Visual design researchers have used this framework to document and investigate affective, cognitive, and behavioral consumer responses to visual design[26]. Briefly, the Context of Consumption Framework suggests cognitive responses to product design involve the processing of how the design influences individuals' perceptions of the product's characteristics, the aesthetics of the packaging, and their own identity. Affective responses include emotional responses to how the product will satisfy a need or desire, feelings about the social positioning of the product, and interest. Cognitive and affective responses influence behavioral responses, leading to product approach or avoidance. Research suggests this framework is appropriate for studies of cigarettes[27]. This framework informs our study. To address these gaps and guided by theory, we conducted two separate experiments. In both, we used an experimental design in combination with a discrete-choice task, where research participants

selected one pack per randomized set of choices.

First, we aimed to examine the role of subtle changes to a product on consumer choices. Specifically, we examined subtle changes in logo size, pack dimensions, and color saturation. Our first experiment's stimuli were intentionally selected to test the role of subtle changes to assess if FDA was fully leveraging its powers to protect the public. At the time we designed our research, subtle changes such as logo size and color saturation were already being identified by FDA as potentially not worthy of regulation – both logo size and color saturation were specifically referenced by the FDA in its second edition draft guidance for industry as 'examples that may not result in a distinct product'[28]. Pack dimensions were noted to be of interest by FDA, but the evidence cited by FDA has the limitations noted above and focused largely on very noticeable changes to packaging[29]. Thus, experiment one was designed to provide evidence regarding consumer choices in relation to small changes to packaging.

Second, we aimed to link changes in pack design to adult smokers' perceptions of harm, appeal, and match with one's own style. Specifically, we examined color hue, designs with natural/organic imagery, and color saturation. Our second experiment's stimuli were intentionally selected to test the role of changes that FDA's early guidance suggest would yield a new product as 'examples that may result in a distinct product'[28]. Given the importance of color in tobacco packaging, we also sought to further explore the role of color saturation. Thus, experiment two was designed to provide evidence regarding consumer choices in relation to large changes to packaging.

METHODS

Study design

To address our study aims, for each experiment we utilized a within-subjects design; specifically, we used a balanced lattice design with 64 different pack designs (Plan 10.5 in Cochran and Cox)[30] in combination with discrete-choice tasks. Discrete-choice tasks[31] ask participants to choose between presented options. As such, they provide a closer proxy for behavioral decision making than do ratings, and they can be used to disentangle what factors contribute to choices. Following a power calculation and based on a prior study[32], we planned for 275 respondents in each experiment. Using the advanced block randomization feature of Qualtrics, each participant was randomized to view one of nine repetitions containing eight blocks of eight different packs. In each block, the participant completed a discrete-choice task. We also randomized the order in which the blocks were presented. Thus, each participant made eight choices in total for each dependent variable. Supplementary file Figure S1 shows the design of the study. Balanced lattice designs are ideal for experiments with many different variations[30].

Design of stimuli

To develop our stimuli, we drew upon focus groups with US adult smokers about cigarette pack designs[33]. A professional graphic designer with training in product packaging design then iteratively developed a unique brand for our study, Glacier, after reviewing existing packs on the market. Figure 1 shows the pack's design.

For the first study on newness, we created four packs of different dimensions, four logo sizes, and four color saturation levels. We also created an 'average' pack (Figure 1). Variations were developed by the graphic designer to produce differences that were the smallest change to make a distinguishable difference. For example, color saturation was developed with a light blue and a dark blue and the designed used a gradient tool to transition between them. In Cyan, Magenta, Yellow, and Key (CMYK) values, the color

Figure 1. Study 1 reference pack

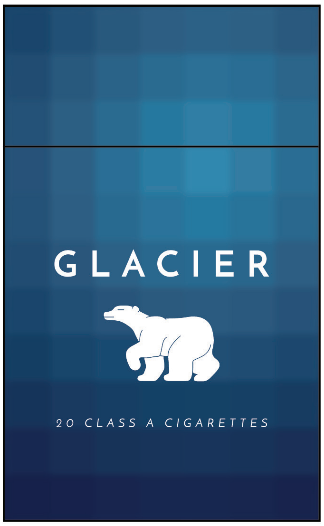

Figure 2. Study 2 imagery examples

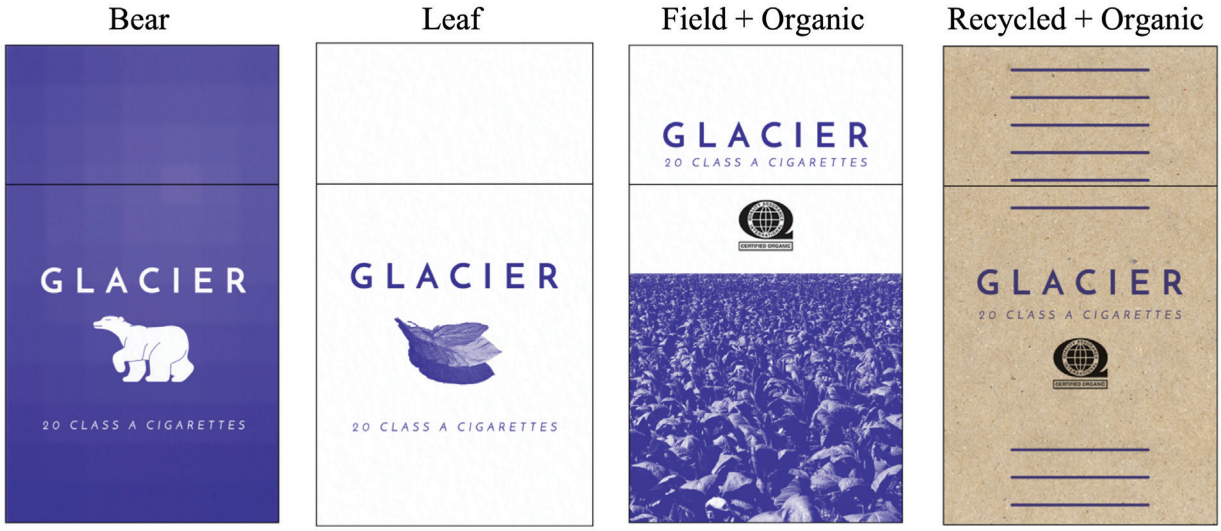

saturation ranged from: Light Blue (20,0,0,20) and Dark Blue (80,60,0,20); to Light Blue (100,10,0,32) and Dark Blue (100,94,0,90); with the average pack (Figure 1) having values of Light Blue (53,10,0,32) and Dark Blue (100,94,0,50). Logo sizes ranged from: 0.4421 × 0.2892 inches to 1.0315 × 0.6749 inches; with the average pack having values of 0.7368 × 0.4821 inches.

For the second study, we created eight color hues (blue, gold, green, light blue, light green, orange, purple, and red), four levels of natural imagery (standard pack from the first study, a leaf logo, a field of tobacco leaves and organic logo, and a pack with the appearance of unbleached recycled paper and an organic symbol), and two levels of color saturation. Figure 2 shows the imagery. All stimuli are available in our institutional repository [https://dataverse.unc.edu/dataverse/R03CA212542].

Sample and recruitment

To recruit participants, we utilized panel provider Qualtrics Research Services, which does not maintain its own survey panel but contracts with other panel providers and, using a proprietary algorithm, selects participants from across multiple panels. Qualtrics's survey panel service has pre-screened and enroled research participants, conducts recruitment, and pays incentives to their survey panel participants. Specifically, we used the following quota sampling requirements to ensure a diverse study population: a 50–50 gender split based on sex assigned at birth, ≥33% have <4 years higher education, ≥33% identifies as sexual or gender minority, ≥15% identifies as Black/African American, and ≥15% identifies as Hispanic/Latino. We oversampled sexual or gender minority adults due to higher prevalence of smoking among lesbian, gay, and bisexual adults. Participants could participate in only one of the two surveys, i.e. we used non-overlapping samples in the two experiments. Beyond the quota sampling, we did not require that the two surveys have the same mix of participants. Participants were only eligible if: 1) they were using a computer (i.e. participants using mobile devices were not eligible), 2) they were aged ≥18 years, 3) they reported not being red-green color blind, 4) they lived in the US and spoke English, and 5) they had smoked 100 cigarettes in their lifetime and currently smoked every day or some days. To improve data quality, Qualtrics also excluded participants who failed attention checks or who completed the survey in less than half of the median time to complete it during its soft launch. Qualtrics fielded the experiments concurrently from 14 November to 18 December 2018. Table 1 shows the participant characteristics.

Measures

Demographic variables

We assessed age with 'What is your age?' and provided a text response box. We assessed smoking status by asking if participants had smoked 100 cigarettes

in their life. We then asked: 'Do you now smoke cigarettes every day, some days, or not at all?'. We used one item from the Fagerström Test for Nicotine Dependence[34]: 'How soon after you wake up do you smoke your first cigarette?'. To have a binary variable for quota sampling, we asked: 'What sex were you assigned at birth, on your original birth certificate?' with options of male and female, as no US state provides for other options on birth certificates at the time of our participants' birth. We asked participants to identify 'Which one or more of the following would you say is your race?' and 'Are you Hispanic, Latino, or Spanish origin?'. To assess educational attainment, we asked: 'What is the highest grade or year of school you completed with five response options' (Grades 1–8, 9–11, 12 or GED, some college or technical school, and college graduate).

Table 1. Participant characteristics by study, 2018

Characteristics	Study 1 n=285 n (%)	Study 2 n=284 n (%)
**Smoking frequency**		
Every day	210 (73.7)	232 (81.7)
Some days	75 (26.3)	52 (18.3)
Usually smoke menthol	142 (49.8)	144 (50.7)
**Time to first cigarette after waking** (minutes)		
>60	79 (27.7)	63 (22.2)
31–60	52 (12.2)	49 (17.3)
6–30	92 (32.3)	90 (31.7)
≤5	62 (21.8)	81 (28.5)
**Age** (years), mean ± SD	46.9 ± 14.3	48.4 ± 16.3
**Sex assigned at birth**		
Female	137 (49.1)	143 (50.4)
Male	145 (50.9)	141 (49.6)
**Race/ethnicity***		
American Indian or Alaska Native	10 (3.5)	6 (2.1)
Asian	8 (2.8)	8 (2.8)
Black or African American	43 (15.1)	44 (15.5)
White	226 (79.3)	209 (73.6)
Other	8 (2.8)	24 (8.5)
Hispanic, Latino, or Spanish origin	43 (15.1)	43 (15.1)
**Sexual orientation**		
Straight or heterosexual	193 (67.7)	186 (65.5)
Gay or lesbian	45 (15.8)	52 (18.3)
Bisexual	47 (16.5)	34 (12.0)
**Educational level**		
<4 years of college	111 (38.9)	186 (65.5)
≥4 years of college	174 (61.1)	98 (34.5)

*Multiple choices. Percentages do not total to 100 due to sporadic missing values.

Study 1: Dependent variable

After consent and screening questions, we gave participants the following prompt: 'On the following pages, we would like you to imagine that you have gone into a store to buy this pack of cigarettes (Figure 1 was displayed). If it was not available, but there were similar packs, we would like you to tell us which one you would buy instead. Please assume the pack styles you will see are the only ones available and they all sell for the same price. There are 8 sets of packs for you to compare'. After this screen, participants made the eight choices, one per screen. Each had the following prompt: 'Imagine you went to the store to buy this pack of cigarettes (Figure 1 was displayed). If it was not available, but there were some very similar packs, if these were the packs you had to choose from, which one would you be most likely to buy? They are all the same price'. Participants then selected a pack and moved to the next screen.

Study 2: Dependent variables

We used the following instructions prior to the discrete-choice task: 'We are going to show you 8 sets of cigarette packs. In each set of packs, we will ask you to choose a pack that seems the least harmful to your health, that seems like it would best match your style, and that seems most appealing to you. They are all the same price'. For each choice, we reiterated the instructions: 'Please imagine you went to the store to buy cigarettes. The only options available were the packs below. They are all the same price'. We then requested: 1) 'Select the one that seems the least harmful to your health', 2) 'Select the one that seems most appealing to you'; and 3) 'Select the one that seems like it would best match your style'. Participants could select the same or different packs for each request.

Statistical analysis

For data management and descriptive statistics, we used SPSS v. 26 (IBM, Chicago, IL). We restructured data so each choice was a row. To analyze the

choice experiments, we used Latent Gold Software v 5.1 (Statistical Innovations, Arlington, MA), which was designed for the analysis of discrete-choice experiments. We report choice parameters, 95% confidence intervals calculated with robust sandwich standard errors, and associated Wald tests, which allow us to present comparisons of levels (e.g. different colors) within attributes (e.g. color hue) of the design. We conducted pairwise comparisons between each package attribute's levels, which are presented in our institutional repository [https://dataverse.unc.edu/dataverse/R03CA212542]. We also report a measure of relative importance. The estimated parameters from the discrete-choice model are utilities from the field of economics (i.e. values established based on preferences). The parameter is not inherently interpretable in itself; however, the estimates indicate the relative influence of the level of the attribute on choices. The importance measure of an attribute is the difference between the maximum utility and minimum utility for its levels. It can also be expressed as a relative measure to other attributes[35], which can be interpreted as the weight of the given attribute in choices made by participants.

RESULTS

Study 1

When tasked with picking the most similar package to an average package, package dimensions, color saturation, and logo size significantly predicted choices with Wald tests of 100.0 and $p<0.001$, 52.5 and $p<0.001$, and 18.3 and $p<0.001$, respectively. The relative importance of each characteristic indicated that subtle changes to pack dimensions had the greatest relative importance (55% of participant choice was driven by dimensions), followed by color saturation (33%). Logo size had the lowest relative importance (12%). Table 2 shows model estimates, which indicate greater or lower preference in participant choices, and if their 95% confidence interval crosses zero. Participants were unlikely to think pack 1 (3.3124 × 2.5 inches) was the most similar package, and participants were most likely to select pack 4 (4 × 2.125 inches) as most similar. Pairwise comparisons (Supplementary file Table S1) indicate that for each attribute type, the two packs with the most similar characteristics as the average pack were selected with no significant difference between each other, indicating that pack characteristics most like the average pack were selected similarly by participants. Thus, participants noticed and distinguished between the more extreme of our subtle design changes; changes in dimensions and color saturation had the biggest influence on the discrete-choice task.

Study 2

Appeal

For the most appealing choice task, color hue, design, and color saturation were each significant predictors of choices with Wald tests of 120.81 and $p<0.001$, 49.30 and $p<0.001$, and 17.30 and $p<0.001$, respectively. The relative importance of each characteristic indicated that color hue was the most important characteristic (50%), followed by the design (38%), and color saturation (12%). Table 3 shows a preference for green packages and the pack 1 (bear) and pack 2 designs (leaf). In pairwise comparisons (Supplementary file Table S2), there were significant differences between most different color hues, and pack 1 (bear) and pack 2 had significant differences between pack 1 and packs 3 (field + organic) and 4 (recycled + organic) and pack 2 and pack 4.

Table 2. Study 1 package attribute and pairwise comparisons for discrete-choice task of picking most similar package to an 'average' package, 2018 (n=285 cases, n=2253 replications)

Attributes		Estimate	95% CI
Package dimensions (inches)			
1	3.3124 × 2.5	-0.92	-1.10 – -0.73
2	2.5 × 2	0.22	0.09 – 0.34
3	2.3125 × 2.125	0.28	0.14 – 0.41
4	4 × 2.125	0.42	0.28 – 0.56
Color saturation			
1	Lightest	-0.57	-0.73 – -0.41
2	.	0.19	0.09 – 0.28
3	.	0.23	0.13 – 0.33
4	Darkest	0.16	0.04 – 0.28
Logo size			
1	Smallest	-0.07	-0.17 – 0.03
2	.	-0.13	-0.22 – -0.03
3	.	0.16	0.07 – 0.24
4	Largest	0.04	-0.05 – 0.14

Table 3. Study 2 model estimates of attributes by discrete-choice task, 2018 (n=284 cases, n=2263 replications)

Attributes		Choice task					
		Appeal		Least harm		Style	
		Estimate	95% CI	Estimate	95% CI	Estimate	95% CI
Color hue							
1	Blue	-0.67	-0.83 – -0.51	-0.50	-0.73 – -0.47	-0.75	-0.90 – -0.59
2	Gold	-0.37	-0.54 – -0.19	-0.10	-0.25 – 0.06	-0.18	-0.35 – -0.01
3	Green	0.49	0.34 – 0.65	0.17	0.02 – 0.31	0.56	0.40 – 0.71
4	Light blue	0.34	0.20 – 0.49	0.30	0.16 – 0.43	0.28	0.13 – 0.42
5	Light green	0.10	-0.03 – 0.23	0.34	0.19 – 0.48	0.10	-0.03 – 0.23
6	Orange	-0.10	-0.25 – 0.04	-0.05	-0.17 – 0.08	-0.09	-0.23 – 0.06
7	Purple	0.21	0.07 – 0.36	0.13	0.00 – 0.25	0.05	-0.10 – 0.20
8	Red	-0.01	-0.16 – 0.13	-0.19	-0.33 – -0.04	0.02	-0.13 – 0.17
Design*							
1	None (bear)	0.34	0.21 – 0.48	-0.26	-0.42 – -0.10	0.28	0.15 – 0.42
2	Limited (leaf)	0.24	0.11 – 0.37	-0.15	-0.31 – 0.00	0.32	0.18 – 0.46
3	Field + organic	-0.02	-0.18 – 0.13	0.81	0.67 – 0.95	-0.02	-0.18 – 0.13
4	Recycled + organic	-0.56	-0.74 – -0.37	-0.39	-0.58 – -0.21	-0.58	-0.77 – -0.39
Color saturation							
1	Saturated	-0.14	-0.21 – -0.07	-0.05	-0.12 – 0.02	-0.14	-0.21 – -0.07
2	Low saturation	0.14	0.07 – 0.21	0.05	-0.02 – 0.12	0.14	0.07 – 0.21

*Type of natural imagery.

Least harm

Design and color hue were statistically significant predictors of choices with Wald tests of 122.8 and p<0.001, and 101.5 and p<0.001, respectively. Color saturation was not statistically significant, Wald test of 1.8 and p=0.18. For the least harmful choice task, participant choices indicated that the relative importance of design (54%) was greatest, followed by color hue (42%) and color saturation (4%). As shown in Table 3, the pack with the field of tobacco leaves and an organic certification, was most commonly chosen in this choice task as indicated by the estimate. Indeed, this was the largest estimate in the model for any choice. Pairwise comparisons (Supplementary file Table S3) show that pack 3 (field + organic) was significantly more likely to be chosen than the standard bear pack (pack 1) and the recycled pack (pack 4), but it was not significantly less likely to be chosen than the leaf pack (pack 2) as least harmful.

Style

For the best matches style discrete-choice task, design, color hue, and color saturation were significant predictors of participant choices with Wald tests of 49.53 and p<0.001, 118.01 and p<0.001, and 16.57 and p<0.001, respectively. Participant choices showed that color hue was most important (52%) followed by design (36%) and color saturation (11%). In pairwise comparisons (Supplementary file Table S4), most differences between color hues were significant, and pack 1 (bear) and pack 2 (leaf) were chosen over packs 3 (field + organic) and 4 (recycled + organic), with significant differences between pack 1 and packs 3 and 4 as well as between pack 2 and pack 4.

Across the different discrete-choice tasks in experiment 2, participants' choices were influenced by the different packaging attributes. Color hue and design exerted the most influence over choices, as expected. There was a preference for the green color hue across the three choice tasks. Regarding design, there were similar patterns of choices between the different designs across the three discrete-choice tasks, except where the task was to select the pack with the least harm, which prompted a clear choice

for the pack featuring a field of tobacco leaves and an organic certification.

DISCUSSION

Principal findings

In our first discrete-choice experiment looking at subtle changes to a cigarette pack, we found that the relative importance of subtle changes to a tobacco product was highest for package dimensions, followed by color saturation. Logo size had the lowest importance. Thus, regulators should be aware that small but noticeable variations in a package are indeed noticed by consumers. More extreme changes to packaging dimensions should be monitored by regulators, given more extreme changes to package dimensions can indicate a new product[29], and package dimensions were a design feature that most influenced our experiment's discrete-choice task. Our results suggest FDA's early guidance[28] not to focus on color saturation and, especially, on logo size was likely warranted. Resources could be directed to assess and monitor other areas of product packaging.

Our second discrete-choice experiment looked at variations by hue, natural/organic imagery, and color saturation across three choice tasks to select: 1) the most appealing, 2) the least harmful, and 3) the best match with one's style. In each of these, hue and natural/organic imagery had the highest relative importance for choices, and color saturation ranked last. Thus, regulators should consider the color hue of packages and the imagery used on them, as these influence consumer choices. FDA's early guidance[28] that such changes could constitute a distinct product were likely warranted. Regulators would do well to monitor these types of changes to product packaging when legally authorized to do so given their influence on choices relating to appeal, harm, and style.

Study results in context

In Study 1, we found that subtle changes to a product package are noticeable to adult smokers and that dimensions and color saturation were more important to the choices made by participants than logo size. Given the existing literature showing that manufacturers of new products may want to extend an existing product's branding rather than replace it with something new[3-5], these findings provide early evidence that regulators should consider subtle changes in product packaging – especially regarding dimensions.

Our finding that changes to cigarette packages influenced consumers' choices is consistent with theory[6,26]. It is also consistent with prior research into tobacco industry documents, which show the tobacco industry carefully calibrates packaging to reach specific profiles of consumers and to intimate differences in product characteristics[8,9]. Color is also used to convey information about the product inside the pack[10]; indeed, previous research shows color has been used to evade bans on text descriptors such as 'light' and 'mild' on packages as well as communicate flavor[7]. Packaging can clearly communicate the harms of the product, its appeal, and its relevance for one's own identity. Finally, our findings match the ample evidence for plain packaging regulation[16-21]; however, our findings are useful in the US context given plain packaging is unlikely to survive a court challenge in the US[22]. Our findings, however, are not consistent with the current implementation of regulations, which provide substantial leeway for design changes by the industry[36].

Strengths and limitations

The study strengths of an experimental design, behavioral discrete-choice task, and use of theory-informed measures must be balanced against its limitations. First, the sample we used was based on quota sampling from an online survey panel and may not be generalizable to the population of smokers in the US. However, prior research shows that similar experiments in tobacco research tend to be generalizable from such panels[37]. Second, our research included only adult smokers. Future work should consider the impact of designs on youth and non-smokers. Third, our experimental discrete-choice task has strong internal validity; however, real world choices take place in a much more complex environment with marketing, word of mouth, brand identity, price promotions, and other influences on choice tasks. Thus, the internal validity of our study must be balanced against its more limited ecological validity. Fourth, we designed our stimuli with a professionally trained graphic designer. However, packaging designs used by the tobacco industry are carefully calibrated by extensive formative research[8]; our approach may have attenuated the influence of

designs. We did not test an exhaustive number of packaging features; future work should expand to other potential packaging characteristics. Finally, since our study was designed and funded, the U.S. FDA has since lost a court case that limits its ability to assess changes in labeling for the purpose of determining a distinct tobacco product[38]. We report the results of our experiments so they will be available if future court decisions open the possibility of greater regulation of packaging changes.

CONCLUSIONS

Among adult smokers in the US, changes to cigarette packaging design are associated with choices about the selection of cigarette packages. They show that even small changes to cigarette packages are noticeable to adult smokers. Packaging designs can influence choices related to health, product appeal, and individual identity. These findings are consistent with the theoretical literature, tobacco industry documents, and prior studies. Marketing researchers and behavioral scientists will likely agree that our findings are consistent with the scientific evidence base and are, effectively, unsurprising. Yet, our findings provide valuable evidence to regulators, who require specific evidence relevant to tobacco products for their work. The scientific evidence needed for regulatory science goes beyond scientific consensus[39]. Our findings add to the available tools of regulators by: 1) being specific to the US, 2) being directly about tobacco products, 3) utilizing a strong experimental design, and 4) leveraging theory-informed measures. In summation, we provide evidence to suggest the importance of regulating the visual design of cigarettes – and we would argue of other tobacco products.

REFERENCES

1. Gray N. Regulation of consumer products: the bizarre case of strawberry jam and cigarettes. Respirology. 2008;13(6):761-763. doi:10.1111/j.1440-1843.2008.01374.x
2. National Center for Chronic Disease Prevention and Health Promotion (US) -Office on Smoking and Health. Smoking-Attributable Morbidity, Mortality, and Economic Costs. The Health Consequences of Smoking—50 Years of Progress: A Report of the Surgeon General. Centers for Disease Control and Prevention (US); 2014. Accessed May 21, 2021. https://www.ncbi.nlm.nih.gov/books/NBK294316/
3. Schoormans JPL, Robben HSJ. The effect of new package design on product attention, categorization and evaluation. J Econ Psychol. 1997;18(2-3):271-287. doi:10.1016/S0167-4870(97)00008-1
4. Person O, Schoormans J, Snelders D, Karjalainen TM. Should new products look similar or different? The influence of the market environment on strategic product styling. Des Stud. 2008;29(1):30-48. doi:10.1016/j.destud.2007.06.005
5. Mugge R, Dahl DW. Seeking the ideal level of design newness: Consumer response to radical and incremental product design. J Prod Innov Manage. 2013;30:34-47. doi:10.1111/jpim.12062
6. Bloch PH. Seeking the ideal form: Product design and consumer response. Journal of Marketing. 1995;59(3):16-29. doi:10.1177/002224299505900302
7. Bansal-Travers M, O'Connor R, Fix BV, Cummings KM. What do cigarette pack colors communicate to smokers in the U.S.? Am J Prev Med. 2011;40(6):683-689. doi:10.1016/j.amepre.2011.01.019
8. Wakefield M, Morley C, Horan JK, Cummings KM. The cigarette pack as image: new evidence from tobacco industry documents. Tob Control. 2002;11 Suppl 1(Suppl 1):I73-I80. doi:10.1136/tc.11.suppl_1.i73
9. Difranza JR, Clark DM, Pollay RW. Cigarette package design: opportunities for disease prevention. Tob Induc Dis. 2002;1(2):97-109. doi:10.1186/1617-9625-1-2-97
10. Lempert LK, Glantz S. Packaging colour research by tobacco companies: the pack as a product characteristic. Tob Control. 2017;26(3):307-315. doi:10.1136/tobaccocontrol-2015-052656
11. Bansal-Travers M, Hammond D, Smith P, Cummings KM. The impact of cigarette pack design, descriptors, and warning labels on risk perception in the U.S. Am J Prev Med. 2011;40(6):674-682. doi:10.1016/j.amepre.2011.01.021
12. Ling PM, Glantz SA. Using tobacco-industry marketing research to design more effective tobacco-control campaigns. JAMA. 2002;287(22):2983-2989. doi:10.1001/jama.287.22.2983
13. Carpenter CM, Wayne GF, Connolly GN. Designing cigarettes for women: new findings from the tobacco industry documents. Addiction. 2005;100(6):837-851. doi:10.1111/j.1360-0443.2005.01072.x
14. Lempert LK, Glantz SA. Implications of Tobacco Industry Research on Packaging Colors for Designing Health Warning Labels. Nicotine Tob Res. 2016;18(9):1910-1914. doi:10.1093/ntr/ntw127
15. Epperson AE, Henriksen L, Prochaska JJ. Natural American Spirit Brand Marketing Casts Health Halo Around Smoking. Am J Public Health. 2017;107(5):668-670. doi:10.2105/AJPH.2017.303719
16. McNeill A, Gravely S, Hitchman SC, Bauld L, Hammond D, Hartmann-Boyce J. Tobacco packaging design for reducing tobacco use. Cochrane Database Syst Rev. 2017;4(4):CD011244. doi:10.1002/14651858.CD011244.pub2
17. Wakefield MA, Germain D, Durkin SJ. How does increasingly plainer cigarette packaging influence

adult smokers' perceptions about brand image? An experimental study. Tob Control. 2008;17(6):416-421. doi:10.1136/tc.2008.026732

18. Stead M, Moodie C, Angus K, et al. Is consumer response to plain/standardised tobacco packaging consistent with framework convention on tobacco control guidelines? A systematic review of quantitative studies. PLoS One. 2013;8(10):e75919. doi:10.1371/journal.pone.0075919
19. Freeman B, Chapman S, Rimmer M. The case for the plain packaging of tobacco products. Addiction. 2008;103(4):580-590. doi:10.1111/j.1360-0443.2008.02145.x
20. Moodie C, Angus K, Stead M. Consumer Response to Standardized Tobacco Packaging in the United Kingdom: A Synthesis of Evidence from Two Systematic Reviews. Risk Manag Healthc Policy. 2021;14:1465-1480. doi:10.2147/RMHP.S272259
21. Smith CN, Kraemer JD, Johnson AC, Mays D. Plain packaging of cigarettes: do we have sufficient evidence?. Risk Manag Healthc Policy. 2015;8:21-30. doi:10.2147/RMHP.S63042
22. Gostin LO. Corporate speech and the Constitution: the deregulation of tobacco advertising. Am J Public Health. 2002;92(3):352-355. doi:10.2105/ajph.92.3.352
23. Hammond D. Tobacco packaging and labeling policies under the U.S. Tobacco Control Act: research needs and priorities. Nicotine Tob Res. 2012;14(1):62-74. doi:10.1093/ntr/ntr182
24. Ashley DL, Backinger CL, van Bemmel DM, Neveleff DJ. Tobacco regulatory science: research to inform regulatory action at the Food and Drug Administration's Center for Tobacco Products. Nicotine Tob Res. 2014;16(8):1045-1049. doi:10.1093/ntr/ntu038
25. Kotnowski K, Fong GT, Gallopel-Morvan K, Islam T, Hammond D. The Impact of Cigarette Packaging Design Among Young Females in Canada: Findings From a Discrete Choice Experiment. Nicotine Tob Res. 2016;18(5):1348-1356. doi:10.1093/ntr/ntv114
26. Crilly N, Moultrie J, Clarkson PJ. Seeing things: Consumer response to the visual domain in product design. Des Stud. 2004;25(6):547-577. doi:10.1016/j.destud.2004.03.001
27. Lee JG, Averett PE, Blanchflower T, Gregory KR. Qualitative assessment of a context of consumption framework to inform regulation of cigarette pack design in the U.S. Tob Induc Dis. 2018;16(February). doi:10.18332/tid/82925
28. Food and Drug Administration. Guidance for Industry Demonstrating the Substantial Equivalence of a New Tobacco Product: Responses to Frequently Asked Questions (Edition 2). Accessed May 21, 2021. https://www.regulations.gov/document/FDA-2011-D-0147-0023
29. Portnoy D. Supporting Memorandum: Effect of Tobacco Product Package Shape on Consumer Perceptions, Initiation, and Cessation. U.S. Food and Drug Administration; 2016. Accessed May 21, 2021. https://www.fda.gov/media/124678/download
30. Cochran WD, Cox GM. Experimental designs. 2 ed. John Wiley & Sons; 1992.
31. Greene WH. Discrete choice modeling: Palgrave handbook of econometrics. 2009:473-556. doi:10.1057/9780230244405_11
32. Hoek J, Gendall P, Eckert C, Kemper J, Louviere J. Effects of brand variants on smokers' choice behaviours and risk perceptions. Tob Control. 2016;25(2):160-165. doi:10.1136/tobaccocontrol-2014-052094
33. Lee JGL, Averett PE, Blanchflower T, Landi N, Gregory KR. "Their Packaging Has Always Been Like a Power": A Qualitative Study of U.S. Smokers' Perceptions of Cigarette Pack Visual Design Features to Inform Product Regulation. Int J Environ Res Public Health. 2017;14(10):1234. doi:10.3390/ijerph14101234
34. Heatherton TF, Kozlowski LT, Frecker RC, Fagerström KO. The Fagerström Test for Nicotine Dependence: a revision of the Fagerström Tolerance Questionnaire. Br J Addict. 1991;86(9):1119-1127. doi:10.1111/j.1360-0443.1991.tb01879.x
35. Vermunt JK, Magidson J. Latent Gold® Choice 4.0 User's Manual. Statistical Innovations Inc.; 2005.
36. Ehrlich SL, Woodlee JW. Philip Morris USA Inc. v. FDA. Food and Drug Law Institute; 2017:55-62. Accessed May 21, 2021. https://www.fdli.org/wp-content/uploads/2017/05/Top-Cases-2016-17.pdf
37. Jeong M, Zhang D, Morgan JC, et al. Similarities and Differences in Tobacco Control Research Findings From Convenience and Probability Samples. Ann Behav Med. 2019;53(5):476-485. doi:10.1093/abm/kay059
38. Food and Drug Administration. Guidance for Industry; Demonstrating the Substantial Equivalence of a New Tobacco Product: Responses to Frequently Asked Questions (Edition 3). 2016. Accessed May 21, 2021. https://www.regulations.gov/document/FDA-2011-D-0147-0025
39. Berman ML, Kim AE. Bridging the gap between science and law: the example of tobacco regulatory science. J Law Med Ethics. 2015;43(0 1):95-98. doi:10.1111/jlme.12227

ACKNOWLEDGEMENTS
The authors thank Nunzio Landi for graphic design work and Roy Ellis for helpful edits. The authors thank Janet Hoek and Philip Gendall for sharing their discrete-choice survey.

CONFLICTS OF INTEREST
The authors have each completed and submitted an ICMJE form for disclosure of potential conflicts of interest. The authors declare that they have no competing interests, financial or otherwise, related to the current work. J.G.L. Lee, K.F. O'Brien and P.E. Averett report that they received support for this publication from the National Cancer Institute of the National Institutes of Health and Food and Drug Administration Center for Tobacco Products (CTP) under Award Number R03CA212542. J.G.L. Lee also reports payments to his Institution in the past 36 months from Cancer Research UK.

FUNDING
Research reported in this publication was supported by the National Cancer Institute of the National Institutes of Health and Food and Drug Administration Center for Tobacco Products (CTP) under Award Number R03CA212542. The content is solely the responsibility of the authors and does not necessarily represent the official views of the National Institutes of Health or the Food and Drug Administration. The funder had no role in the design of the study or in data collection, data analysis, interpretation, and writing of the manuscript.

ETHICAL APPROVAL AND INFORMED CONSENT

The East Carolina University and Medical Center IRB approved the study protocol (No. 16-001200 on August 17, 2016). The participants of the study gave informed consent.

DATA AVAILABILITY

The data supporting this research are available from the following source: https://dataverse.unc.edu/dataverse/R03CA212542

AUTHORS' CONTRIBUTIONS

JGLL and KRG originated the study. KFO conducted the analyses. GS advised on the design of product packaging. JGLL, KFO, TMB, and KRG drafted the manuscript. All authors provided critical feedback, edited the manuscript, and approved the final version.

PROVENANCE AND PEER REVIEW

Not commissioned; externally peer reviewed.
